# The cost, survival, and quality‐of‐life implications of guideline‐discordant imaging for prostate cancer

**DOI:** 10.1002/cnr2.1468

**Published:** 2021-06-17

**Authors:** Aaron N. Winn, Matthew Kelly, Shannon Ciprut, Dawn Walter, Heather T. Gold, Steven B. Zeliadt, Scott E. Sherman, Danil V. Makarov

**Affiliations:** ^1^ School of Pharmacy Medical College of Wisconsin Milwaukee Wisconsin USA; ^2^ Cancer Center Medical College of Wisconsin Milwaukee Wisconsin USA; ^3^ Department of Urology New York University School of Medicine New York USA; ^4^ Department of Population Health New York University School of Medicine New York USA; ^5^ VA New York Harbor Healthcare System New York USA; ^6^ Robert F. Wagner Graduate School of Public Service New York University New York USA; ^7^ Health Services Research and Development Department of Veterans Affairs Medical Center Seattle Washington USA; ^8^ Fred Hutchinson Cancer Research Center Seattle Washington USA; ^9^ Perlmutter Cancer Center New York University School of Medicine New York USA

**Keywords:** cancer care, costs, cost‐effectiveness, prostate cancer, simulation

## Abstract

**Background:**

National Comprehensive Cancer Network (NCCN) guidelines for incident prostate cancer staging imaging have been widely circulated and accepted as best practice since 1996. Despite these clear guidelines, wasteful and potentially harmful inappropriate imaging of men with prostate cancer remains prevalent.

**Aim:**

To understand changing population‐level patterns of imaging among men with incident prostate cancer, we created a state‐transition microsimulation model based on existing literature and incident prostate cancer cases.

**Methods:**

To create a cohort of patients, we identified incident prostate cancer cases from 2004 to 2009 that were diagnosed in men ages 65 and older from SEER. A microsimulation model allowed us to explore how this cohort's survival, quality of life, and Medicare costs would be impacted by making imaging consistent with guidelines. We conducted a probabilistic analysis as well as one‐way sensitivity analysis.

**Results:**

When only imaging high‐risk men compared to the status quo, we found that the population rate of imaging dropped from 53 to 38% and average per‐person spending on imaging dropped from $236 to $157. The discounted and undiscounted incremental cost‐effectiveness ratios indicated that ideal upfront imaging reduced costs and slightly improved health outcomes compared with current practice patterns, that is, guideline‐concordant imaging was less costly and slightly more effective.

**Conclusion:**

This study demonstrates the potential reduction in cost through the correction of inappropriate imaging practices. These findings highlight an opportunity within the healthcare system to reduce unnecessary costs and overtreatment through guideline adherence.

## 1 INTRODUCTION

National Comprehensive Cancer Network (NCCN) guidelines for incident prostate cancer staging imaging have been widely circulated and accepted as best practice since 1996.^1^ Despite these clear guidelines, inappropriate imaging of men with prostate cancer remains prevalent.^2^ While imaging may yield useful additional information for some patients, for many imaging has a low probability of influencing treatment choices, and therefore, its harms outweigh its benefits.^3^, ^4^ Harms include emotional distress, wasteful spending, and increased cancer risk secondary to radiation exposure. On the other hand, underuse, or failure to provide appropriate imaging, can lead to delays in diagnosis, inadequate disease staging, and inappropriate treatment among patients who truly require care. The staging of incident prostate cancer is an ideal scenario to study appropriate imaging use, given the prevalence of guideline‐discordant imaging and its cost to the healthcare system.

To understand changing population‐level patterns of imaging among men with incident prostate cancer, we created a simulation model based on existing literature and incident prostate cancer cases from the SEER‐Medicare database. We believe a comprehensive understanding of changing imaging patterns, based on a nationally representative, adequately staged population, has the best chance to provide actionable suggestions for improvement of prostate cancer care.

## 2 METHODS

Simulation modeling allows researchers to ask a series of what‐if questions to understand how a population's life expectancy, quality of life, and health care spending could be impacted by changing how care is provided. When creating a simulation model, prior work has documented that transparency of the model aides is policy makers and clinicians.^5^, ^6^ Therefore, we primarily used freely available data (SEER data) to project survival as well as results from other studies which document how survival, quality of life and costs are impacted by imaging. The National Cancer Institute Surveillance, Epidemiology, and End Results (SEER) program gathers data on cancer site, stage, and histology for over 25% of the Medicare population living in the SEER reporting areas.^7^, ^8^

The model allowed us to explore how this cohort's survival, quality of life, and Medicare costs would be impacted by making imaging consistent with guidelines compared to current practices. To create a cohort of patients, we identified incident prostate cancer cases from 2004 to 2009 that were diagnosed in men aged 65 and older from SEER. Additionally, these data provided important clinical variables measured at time of diagnosis, which allowed patients to be stratified into distinct NCCN risk groups. Consistent with prior research, we classified patients as high risk if they had prostate‐specific antigen (PSA) greater than 20 ng/ml, Gleason score greater than 7 or stage T3 or greater. Otherwise, men were considered low risk.^9^, ^10^

### 2.1 Overall survival

We used data from SEER to analyze the survival for men using multivariable parametric proportional hazard survival models from the time of diagnosis until death or censoring. Models were adjusted for age at diagnosis as a categorical variable (65–70, 71–75, 76–80, 81–85, 85+), stage at diagnosis (T1, T2, T2a, T2b, T2c, T3, and T4), PSA, and Gleason score. We determined the appropriate functional form (Weibull, Gompertz, or exponential) based on the models' Akaike Information Critera (AIC) and Bayesian information criterion (BIC), which indicated the Gompertz distribution was the best fitting model.

### 2.2 Use of imaging

We used previously published observational research for individuals over age 65 in the United States, which documented use of imaging among incident prostate cancer cases. In incident high‐risk prostate cancer cases in the SEER‐Medicare data, only two‐thirds of the population appropriately received imaging.^9^ Concurrently, in incident low‐risk prostate cancer cases approximately 45% of the population inappropriately received imaging.^9^ In order to allow for correlation between patient factors and use of imaging, we generated the predicted probability of staging based on published odds ratios from a logistic regression.^11^ We generated the constant of the logistic regression to ensure that the population staging was consistent with only two‐thirds of high‐risk men receiving appropriate imaging and 45% of low‐risk men receiving inappropriate imaging. Additionally, among low‐risk men who received inappropriate imaging, prior research has shown that 38% received a false‐positive test result. Among these men, 43% received subsequent inappropriate imaging; we incorporated this into the model.^3^

### 2.3 Impact of imaging on treatment choice

A previous analysis of incident prostate cancer cases in SEER‐Medicare data indicated that among men whose disease is metastatic, 94% will pursue systemic therapy, 5% radiation, and 1% surgery.^12^ Among men who are high‐risk but whose metastatic status is unknown or whose disease is known to be localized, prior research has found that 36% will pursue systemic therapy, 42% radiation, 6% surgery, and 16% will pursue observation.^13^ For men whose disease is low risk and it is either unknown if they are metastatic or known that their disease is localized, prior research has documented that 6% will pursue systemic therapy, 52% radiation, 18% surgery, and 23% will receive no treatment/observation (Figure S1).^14^

### 2.4 Impact of treatment choice on survival

There are some men who do not receive imaging but have metastatic cancer, which is not known at diagnosis. For these men, we assumed that the metastatic disease will be identified within 3 months of diagnosis, and subsequently their treatment approach will change. However, in the time period when the severity of the patient's disease is not known, we assumed that there was an increased risk of death. We modeled this assuming a hazard rate of two in this three‐month period. This was a conservative assumption and biased the results toward the null. We did not assume that imaging would have any other impact on treatment choice or survival.

### 2.5 Utility of treatments (quality of life)

We used previously published studies to quantify a patient's utility of particular health states^15^, ^16^ as shown in Table 1. If patients were in multiple health states simultaneously, such as receiving surgery and having metastatic disease, then we used the minimum method to select the health state with the worst quality of life.

**TABLE 1:** Assumptions

	Base case	Range	Distributional parameters	Source
Treatment choices				
Metastatic known				
Observation	0%	0	Dirichlet (0, 47, 195, 3827)	^12^
Surgery	1%	0.5–1.5%		^12^
Radiation	5%	2.5–7.5%		^12^
Systemic therapy	94%	91–97%		^12^
High‐risk, localized, or metastatic unknown				
Observation	16%	8–24%	Dirichlet (245, 92, 644, 553)	^13^
Surgery	6%	3–9%		^13^
Radiation	42%	21–63%		^13^
Systemic therapy	36%	18–54%		^13^
Low‐risk, localized, or metastatic unknown				
Observation	23%	11.5–34.5%	Dirichlet (956, 758, 2143, 257)	^14^
Surgery	18.4%	9.2–27.6%		^14^
Radiation	52.1%	28.2–76.1%		^14^
Systemic therapy	6.3%	3.2–9.5%		^14^
Hazard rate until incidental diagnosis of metastatic disease	2.0	1.4–2.8	Lognormal (2.0, 0.24)	Assumption
Quality of life utilities				
Observation	0.97	0.955–0.985	Beta (32.36, 1.00)	^15^
Radiation 0–2 months	0.73	0.60–0.865	Beta (2.97, 1.10)	^15^
Radiation 3–12 months	0.78	0.50‐0.85	Beta (3.77, 1.06)	^15^
Surgery 0–2 months	0.67	0.50–0.85	Beta (2.36, 1.16)	^15^
Surgery 3–12 months	0.77	0.50–0.85	Beta (3.58, 1.07)	^15^
Localized disease, systemic therapy 0–12months	0.77	0.50–0.85	Beta (3.58, 1.07)	^15^
Post‐recovery period after surgery, radiation, or systemic therapy, over 12 months	0.95	0.93–1.00	Beta (19.05, 1.00)	^15^
Metastatic disease	0.80	0.70–0.90	Beta (4.20, 1.05)	^16^
Treatment specific costs				
Imaging	409	205–614	Gamma, (1, 409)	^17^
Radiation	23 145	11 573–34 718	Gamma (1, 23 145)	^18^
Surgery	28 507	14 254–42 761	Gamma (1, 28 507)	^18^
Systemic therapy	77 035	38 518–115 553	Gamma (1, 77 035)	^19^
Overall costs				
Annual costs, not last year of life	2769	1385–4154	Gamma (1, 2769)	^20^
Last year of life cost	43 718	21 859–65 577	Gamma (1, 43 718)	^20^

### 2.6 Costs

Costs assumptions came from multiple studies of prostate cancer (Table 1).^18^, ^19^, ^20^ For the costs of specific treatment choices, we assumed that they accrued consistently across the year after diagnosis. For men that changed treatments due to detection of an unknown metastatic cancer, we assumed that they had the costs without “known metastatic status” for the first 3 months and then the monthly cost of treatment knowing they were metastatic for the remaining 9 months. For the years following the treatment year (the first year following diagnosis), we assumed that the costs would be similar despite initial treatment choice, and that the last year of life induced higher expenditures.^20^ All costs were converted to 2017 U.S. dollars using the Consumer Price Index.^21^

### 2.7 Scenario

We simulated the status quo of widespread inappropriate imaging and compared it to the optimal scenario where only individuals with high‐risk prostate cancer received imaging and individual with low‐risk prostate cancer did not.

### 2.8 Microsimulation model implementation

Using the SEER‐based survival model, we created a monthly state transition, microsimulation model that forecasted individual‐level survival, quality of life, and healthcare costs. The population we simulated were 66 366 SEER patients for whom we duplicated each person 20 times to generate the sample of 1 327 320 individuals that we simulated. For each of these 1.3 million individuals, we examined their outcomes whether or not they received guideline‐concordant imaging. Using these individuals ensured that correlation and covariance between clinical factors were retained in simulations. We categorized patients as high or low risk, based on information recorded in the SEER data. We then determined whether they did or did not receive imaging based on current practice patterns reported in the literature as detailed above. We forecasted survival based on age, cancer stage, PSA, and Gleason score. As previously described, for patients who had metastatic disease but did not know this due to lack of imaging, we applied a hazard rate of 2 for survival for the first 3 months following diagnosis. After the 3 months, if a patient was still alive, he then had standard survival based on his demographic and clinical characteristics. We then assigned a quality‐of‐life value (ie, utility) based on the treatment he underwent and whether he had metastatic disease. Additionally, we assigned costs based on whether a patient underwent imaging and the treatment he received. For low‐risk men who received imaging and had a false positive, and then received additional imaging, costs were accrued for both imaging procedures. All costs, life years, and quality of life are discounted at 3%. We conducted the model and analyses using Stata 16.0 (Stata Corp, College Station, TX).

### 2.9 Assessing uncertainty

One‐way sensitivity analysis: we assessed model robustness using one‐way deterministic sensitivity analysis, in which we estimated the net health benefit and willingness to pay (WTP) was $100 000 per quality‐adjusted life year (QALY) gained. To implement this, we sequentially changed estimates for each model parameter, while holding all other parameters constant. We maintained a clinically reasonable range of values, from 50% plus or minus the baseline value (Table 1). For proportions greater than half, we took 50% plus or minus 1 minus the baseline value. We conducted a probabilistic analysis to assess higher order uncertainty. We incorporated uncertainty around model parameters w into the model using probability distributions (Table 1 for a full listing of distributions). Types of treatment were modelled using a Dirichlet distribution, and quality‐of‐life estimates were modelled using a beta distribution. Costs were modelled using gamma distributions with the assumption that the mean is equal to the SE. To generate the predicted probabilities of being staged, we used the parameter confidence intervals reported from the logistic regression^11^ previously referenced. The cohort was simulated 1000 times. We generated bootstrap replications for each of the 1000 simulations and re‐estimated the survival model with the bootstrapped sample. The results of the probabilistic analysis are presented as scatter plots showing the incremental‐cost effectiveness for each iteration and as the cost‐effectiveness acceptability curve (CEAC). The CEAC shows the probability that reducing imaging is cost‐effective relative to the status quo with different levels for the WTP for one quality‐adjusted life‐year.

## 3 RESULTS

Base Case: When only imaging high‐risk men compared to the status quo, we found that the population rate of imaging decreases from 53% to 38%, and discounted average per‐person spending on imaging drops from $236 to $157 (Table 2). We also found that guideline‐concordant imaging resulted in men with metastatic cancers being much more likely to receive initial systemic therapy (72 vs. 94%) instead of starting on a different therapy and switching to a more appropriate therapy once metastatic disease was identified. This earlier detection of metastases resulted in improved initial treatment and slightly lower first‐year treatment costs ($29 993 vs. $29 884, undiscounted). Additionally, we found that improved initial treatment resulted in slightly lower first‐year treatment costs stemming from men with low‐risk disease and unknown metastasis that would have received imaging. These men would have been diagnosed with metastatic disease under the status quo but would not receive imaging in the ideal scenario. This small group of men has an increased risk of mortality until incidental detection.

**TABLE 2:** Simulation results, percentage of men receiving specific services, costs (US$), life years, quality‐adjusted life years, and incremental cost‐effectiveness ratio (2017)

	Status quo imaging	Appropriate imaging	Difference
Received imaging, overall (%)	53	38	−0.15
Received imaging, high risk (%)	66	100	0.34
Received imaging, low risk (%)	45	0	−0.45
Treatment received, overall			
Observation (%)	20	20	−0.00
Surgery (%)	14	14	−0.00
Radiation (%)	47	47	−0.00
Systemic (%)	19	20	0.01
Initial treatment received, high risk			
Observation (%)	15	15	−0.00
Surgery (%)	6	6	−0.00
Radiation (%)	40	39	−0.01
Systemic (%)	39	41	0.02
Initial treatment received, metastatic			
Observation (%)	6	0	−0.06
Surgery (%)	3	1	−0.02
Radiation (%)	19	5	−0.14
Systemic (%)	72	94	0.22
Non discounted costs ($)			
Imaging cost	236	157	−78
first year treatment costs	29 993	29 884	−149
Total lifetime costs	100 035	99 806	−228
Life years	12.0960	12.0961	0.00006
QALYs	11.075	11.075	0.00025
ICER			Cost Saving
Discounted costs ($)			
Total lifetime costs	81 198	80 973	−224
Survival	9.561	9.561	0.00006
QALYs	8.727	8.727	0.00025
ICER			Cost‐Saving

Abbreviations: ICER, incremental cost‐effectiveness ratio; QALYs, quality‐adjusted life years.

The discounted and undiscounted incremental cost‐effectiveness ratios indicated that guideline‐concordant imaging was cost‐saving and slightly more effective compared with current practice patterns. Moreover, the probabilistic sensitivity analysis showed that 98% of iterations reduced costs. Additionally, we found a very small improvement in undiscounted life years (12.0960 vs. 12.0961 life years). This small difference is observed in the probabilistic sensitivity analysis, which found that only 52% of iterations improved health and the remaining 48% iterations reduced QALYs.

**FIGURE 1:**
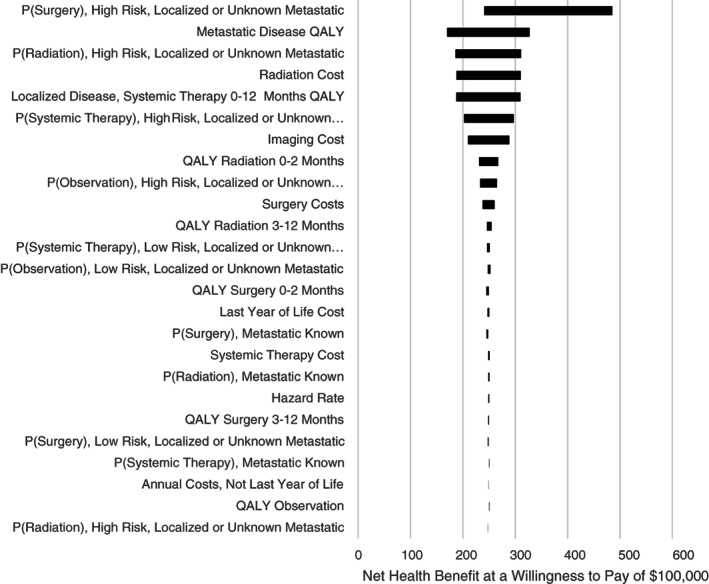
One‐way sensitivity analyses. QALY, quality‐adjusted life year

**FIGURE 2:**
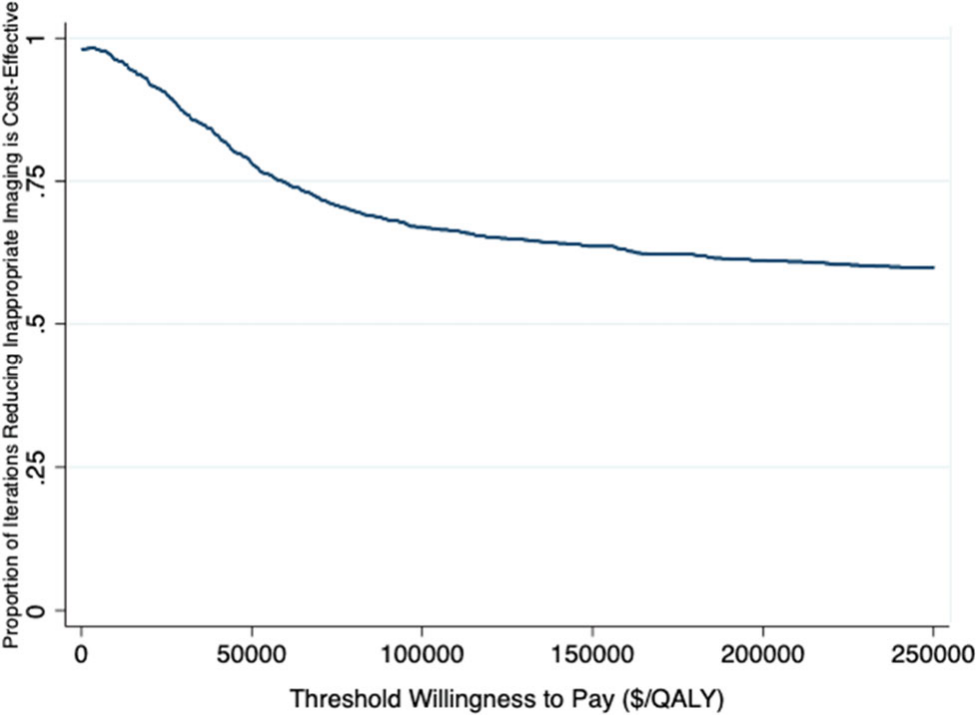
Distribution of incremental cost‐effectiveness ratios (ICERs) for 1000 iterations from the probabilistic analysis of optimal screening compared to the status quo

**FIGURE 3:**
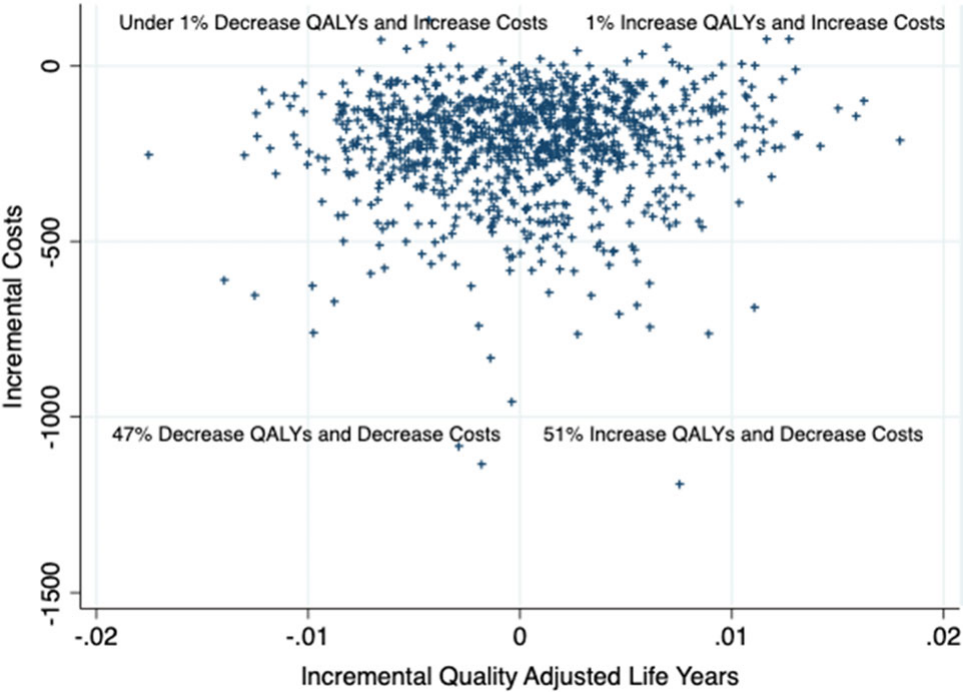
Cost‐effectiveness acceptability curve based on willingness to pay for one extra quality‐adjusted life‐year gained from the probabilistic analysis

All one‐way sensitivity analyses (Figure 1) showed consistent results to the base case. Generally, it appeared that the results were most sensitive to the treatments used for high‐risk men and radiation costs. As shown in Figure 2, the probabilistic analysis had results that spanned all quadrants of the incremental cost‐effectiveness plane, with 51% of iterations improving health and reducing costs, 47% reducing health and costs, under 1% increasing health and costs, and under 1% decreasing health and increasing costs. The CEAC (Figure 3) showed that reducing imaging is the preferred strategy at all WTP thresholds; however, the probability of cost‐effectiveness declined as the WTP increased. This is largely due to iterations where health and costs were both reduced. As the value of a QALY increased, the savings gained mattered less relative to any health lost.

## 4 DISCUSSION

The incremental cost‐effectiveness ratio comparing guideline‐concordant imaging to status quo imaging indicated that guideline‐concordant imaging would be cost saving but would have a limited impact on health. This demonstrates that appropriate reallocation of imaging resources would save the health system money, a situation rarely seen in cancer care.^22^ The spending reduction and QALY benefit of appropriate imaging practice are optimized by limiting guideline‐discordant imaging practices among men with low‐risk prostate cancer. This finding is consistent with clinical intuition and suggests that physicians could reduce waste in the healthcare system by providing care that is consistent with existing clinical guidelines. Medicare and other insurers may want to use reimbursement incentives to motivate clinicians to be consistent with clinical guidelines.^1^

This study has several limitations. First, it is unclear how long men with unknown metastatic cancer would wait until their metastatic disease was detected and how that delay in treatment would impact survival. We assume a 3‐month delay and an increase in probability of death for those 3 months but do not consider any longer‐term effects. Additionally, we do not examine the role of anxiety for men with non‐metastatic disease that have a false‐positive result for metastatic cancer. Prior research in breast cancer staging has found that false positives can have long lasting impacts on mental health, and in turn quality of life.^23^ We also did not fully explore the consequences of each specific treatment for prostate cancer. Finally, the results are based on data from 2004 to 2009 in the SEER‐Medicare database. These data are limited in its applicability as patients comprise a strictly Medicare population aged 65 and up. Our findings may not generalize outside of the NCCN risk groups we used to stratify high and low‐risk patients in the model. In the current staging landscape, there exist novel techniques to stage high‐risk prostate cancer including PSMA PET/CT and NaF PET scans offering alternatives to radionucleotide bone scans. The potential cost/benefit trade‐offs of these nascent imaging tests are an area of interest for future research in prostate cancer staging. The present analysis may actually underestimate the importance of guideline‐concordant staging imaging behaviors in current clinical practices.

The present analysis may actually underestimate the importance of guideline‐concordant staging imaging behaviors in current clinical practices.

Prior work has quantified inappropriate imaging rates as high as 53% for low‐risk prostate cancer patients in a U.S. Medicare setting.^24^ This underscores the strong need and demand for the present analysis as a novel investigation documenting the cost‐benefit trade‐offs. Additionally, in settings with lower rates of inappropriate imaging of low‐risk patients, there usually exists similarly lower rates of appropriate imaging for high‐risk patients.^10^ This suggests that numerous care settings have an opportunity to improve the efficacy and value of the prostate cancer care they provide.

While this model illustrates the excess costs incurred with guideline‐discordant care, it does not account for patient preference. It is possible that imaging could be driven by patient demands in hopes to reduce anxiety; however, a recent qualitative study has found that the decision to pursue imaging is largely driven by physician decision‐making.^25^ Additionally, research has shown that many men with low‐risk disease who receive inappropriate imaging also receive false‐positive test results,^3^ which has been shown to increase anxiety in other cancers.^26^, ^27^

This study demonstrates the potential for cost‐savings through appropriate imaging ordering practices. Cost‐saving interventions are rare, however. A recent review of cost‐effectiveness studies found that only 10% of prostate cancer interventions are cost saving.^22^ The cost reduction shown in our model is modest, suggesting that any intervention to improve imaging guideline adherence should be low cost. One potential solution is the Prostate Cancer Imaging Stewardship (PCIS)^28^ Intervention, a bundle of evidence‐based implementation strategies that target clinician behavior change in order to increase prostate cancer staging imaging guideline concordance. These findings highlight an opportunity within the healthcare system to reduce unnecessary costs and overtreatment through guideline adherence. In current clinical practice, the imaging recommendations and guidelines remain largely unchanged. There are now newer, more expensive technologies for staging, and it is possible that we will be underestimating the importance and value of guideline‐concordant imaging in the field today.

## CONFLICT OF INTEREST

The authors declare that there is no conflict of interest.

## AUTHOR CONTRIBUTIONS

All authors had full access to the data in the study and take responsibility for the integrity of the data and the accuracy of the data analysis. *Conceptualization*, A.W., D.W., H.G., S.Z., S.S., D.M.; *Methodology*, A.W., M.K., D.M.; *Investigation*, A.W., M.K., S.C., D.M.; *Formal Analysis*, A.W., M.K., D.W., H.G., S.Z., S.S., D.M.; *Writing—Original Draft*, A.W., M.K., S.C., D.W., D.M.; *Writing—Review & Editing*, A.W., M.K., S.C., D.W., H.G., S.Z., S.S., D.M.; *Validation*, A.W.; *Data Curation*, A.W., M.K., D.W., H.G., S.Z., S.S., D.M.; *Project Administration*, S.C.; *Supervision*, S.C.; *Funding Acquisition*, D.M.

## ETHICAL STATEMENT

The data underlying this article were freely available de‐identified prostate cancer cases for the years 2004 to 2009 from the SEER‐Medicare database. No institutional approval was required to create this microsimulation model.

## Supporting information


**Figure S1** Prostate cancer screening decision tree

## Data Availability

The data underlying this article were accessed through the SEER‐Medicare database. These data are freely available.
