# Supplementary figures and images for: The cost, survival, and quality‐of‐life implications of guideline‐discordant imaging for prostate cancer
Source: Cancer Rep (Hoboken). 2021 Jun 17;5(2):e1468. doi: 10.1002/cnr2.1468 (PMC8842701; doi:10.1002/cnr2.1468)

Supplemental Materials

Supplemental Figure 1: Prostate Cancer Screening Decision Tree


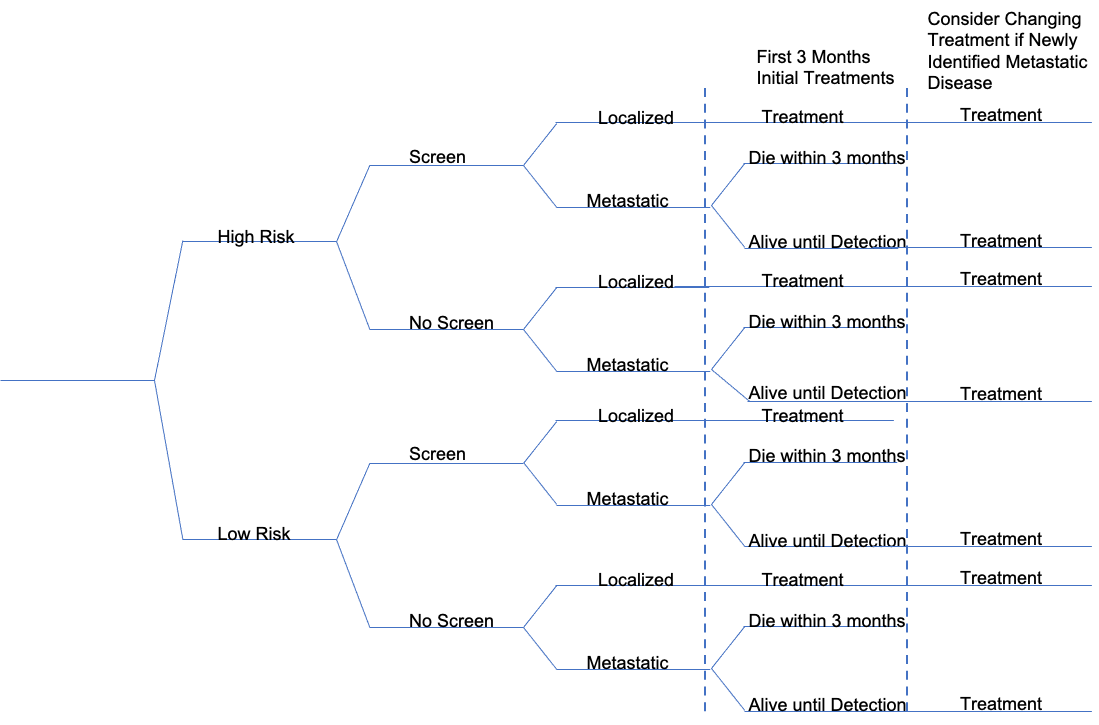

Supplement: Supplementary file 1 — Figure S1 Prostate cancer screening decision tree [file CNR2-5-e1468-s001.docx]
